# Significant delayed aortic dilatation after tetralogy of Fallot repair: a case report

**DOI:** 10.1186/s40792-020-00935-5

**Published:** 2020-07-17

**Authors:** Yosuke Nishimura, Toru Yasutsune, Shohei Shimajiri, Yuki Jinzai, Eigo Ikushima, Takehiro Kishigami, Tomoya Takigawa

**Affiliations:** 1 Department of Cardiovascular Surgery, School of Medicine, University of Occupational and Environmental Health, 1-1, Iseigaoka, Yahatanishi-ku, Kitakyushu, 807-8555 Japan; 2 Department of Surgical Pathology, School of Medicine, University of Occupational and Environmental Health, 1-1, Iseigaoka, Yahatanishi-ku, Kitakyushu, 807-8555 Japan

**Keywords:** Aortic dilatation, Aneurysm, Tetralogy of Fallot

## Abstract

**Background:**

Aortic dilatation may occur in some patients even after complete repair of tetralogy of Fallot (TOF). The progression rate of the aortic diameter is so slow, and the incidence of aortic dissection is so low that it is suspected that frequent imaging of the aorta may not be necessary.

**Case presentation:**

We describe an asymptomatic 41-year-old man with hypertension in whom aortic dilatation was accidentally discovered 39 years after TOF repair. He underwent ambulatory follow-up without any difficulty for 21 years after the repair. Contrast-enhanced computed tomography revealed significant aortic dilatation (maximum diameter of 88 mm at the sinus of Valsalva), and echocardiography revealed severe aortic regurgitation, which seemed to progress during the last 18 years without any evaluation or follow-up. The Bentall procedure was successfully performed using a valved graft, under deep hypothermic circulatory arrest with antegrade cerebral perfusion, and his postoperative course was uneventful. Histopathological examination of ascending aorta specimens revealed severe cystic medial degeneration.

**Conclusions:**

Keeping in mind that a patient with rapid progression of the aortic dilatation after TOF repair exist, periodic follow-up for evaluation of the aorta is essential in patients with TOF.

## Introduction

Total repair of tetralogy of Fallot (TOF) is a safe procedure routinely performed to treat congenital heart disease; the Japanese Association for Thoracic Surgery reported an operative mortality rate of only 0.8% (3 patients) among 375 patients who underwent TOF repair in 2016 [1]. Despite the excellent operative outcomes of TOF repair, many patients develop arrhythmia and reoperations are necessary to treat complications including pulmonary regurgitation or stenosis, or residual ventricular septal defect (VSD) [2]. Aortic root dilatation and aneurysm may occur in patients after TOF repair; these complications are attributable to an intrinsic mechanism or a secondary effect of increased volume overload of the aorta caused by right-to-left shunting [3–6]. Chowdhury et al. reported that approximately 75% of patients with TOF showed histopathological abnormalities of the aortic wall, such as elastic lamellar fragmentation, increased ground substance, medial necrosis, and smooth muscle disarray, since infancy and that these changes were more pronounced in older patients [7]. Although aortic medial degeneration is less extensive and less severe in TOF patients than in those with Marfan syndrome [3, 8], the risk of aortic dilatation is much higher in patients with histopathologically proven aortic abnormalities [7].

Reportedly, the incidence of aortic dilatation defined by various criteria in patients who underwent TOF repair was 14-69% [4–6], and risk factors for aortic dilatation after TOF repair were male sex, smoking, and late age at complete surgical repair [5, 6]. However, the number of patients who underwent aortic operations after TOF repair was very low. A multicenter Japanese study reported the 236 patients (5.9%) of 4010 patients who underwent TOF repair required reoperation, and only 4 patients (0.1%) underwent reoperation to treat aortic dilatation [9]. Egbe et al. reported that among consecutive 453 patients who underwent TOF repair (the mean age was 37 ± 13 years), aortic dilatation occurred in 312 patients (69%), and progressive aortic dilatation (defined as an increase ≧ 2 mm in aortic dimensions) occurred in 40 patients (9%). However, no patient developed aortic dissection, and only 7 patients (1.5%) among them in whom the maximum diameter of aortic root or ascending aorta was more than 45 mm required prophylactic aortic procedures [4]. Kay et al. performed serial studies using magnetic resonance angiography in 55 patients and reported a slow rate of growth of 0.4 ± 0.9 mm/year at the sinuses of Valsalva and 0.1 ± 0.8 mm/year at the ascending aorta [5].

Notably, the incidence of aortic dissection and aneurysm rupture in patients undergoing TOF repair is quite low. To date, no retrospective observational studies have reported aortic dissection in patients undergoing TOF repair [2, 4–6, 9, 10]. A search of the available literature yielded only 4 case reports describing aortic dissection in patients undergoing TOF repair [11–14]. All 4 cases of dissection were Stanford type A and occurred at aortic dimensions of 70 mm or greater. Records obtained from the National Inpatient Sample Database from 2000 to 2014 show that among hospital admissions of 18,353 adults TOF, only 11 (0.06%) were thoracic aortic dissection related admissions (in-hospital mortality was 45%), of which aortic surgical interventions were performed during 8 of the admissions [15]. The risk factors associated with aortic dissection in patients with TOF include male sex, age, and hypertension [15]. Studies have shown that moderately aortic dilatation may occur in patients undergoing TOF repair; however, this condition does not require frequent aortic imaging and early prophylactic surgical intervention because the incidence of aortic dissection in this patient population is very low in contrast to that observed in patients with degenerative and bicuspid aortic valve aortopathies [4, 5, 10].

We describe a patient who underwent TOF repair with significant and rapid aortic root and ascending aorta dilatation of which the maximum diameter reached 88 mm at the level of sinuses of Valsalva during the last 18 years without any periodic follow-up or evaluation.

## Case

**Fig. 1:**
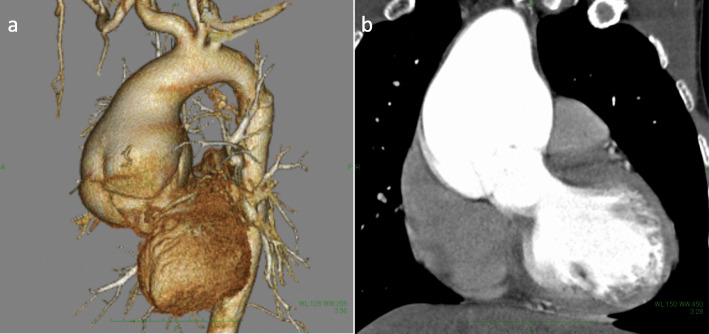
Contrast-enhanced computed tomography scan showing a significantly dilatated aortic root and ascending aorta. Aortic aneurysm was located in the ascending aorta (**a**), and the maximum diameter of the aortic aneurysm is 88 mm at the sinus of Valsalva (**b**)

An asymptomatic 41-year-old man underwent evaluation for employment health assessment and was accidentally discovered to have significant aortic dilatation. He reported a history of total repair of TOF with transannular patching at 2 years of age. Postoperatively, he underwent ambulatory follow-up for 21 years without any difficulty until he discontinued follow-up on his own because he was asymptomatic. Last transthoracic echocardiography (TTE) reports in his pediatric medical records at that time showed only trivial aortic regurgitation (AR) without any aortic root abnormality. On physical examination, he was 173 cm tall, weighed 65.6 kg, and his blood pressure was elevated to 165/60 mmHg; however, he had not received any medication. Contrast-enhanced computed tomography (CT) revealed significant aneurysmal aortic dilatation (maximum diameter of 88 mm at the sinus of Valsalva) (Fig. 1). TTE revealed severe AR, without significant pulmonary regurgitation or residual VSD, and transesophageal echocardiography showed a slight shortening of the noncoronary cusp and poor coaptation of leaflets of the aortic valve at the central portion where a massive AR, which had 0.9 cm^2^ of regurgitant orifice area, could be seen. Cardiac magnetic resonance imaging revealed that significant pulmonary regurgitation flow and residual VSD could not be detected and that right ventricular (RV) ejection fraction was 37%, end-diastolic RV volume index was 201 ml/m^2^. He was referred to our department for surgical treatment of aortic root dilatation and AR.

The procedure was performed through a midline sternotomy, after taping the left femoral artery and vein. Cardiopulmonary bypass was established after femoral arterial and bicaval cannulation. Left ventricular venting was initiated using a venting tube inserted through the right upper pulmonary vein. Exacerbation of AR and onset of ventricular fibrillation were observed after initiation of cooling, necessitating aortic clamping, and antegrade cardioplegic arrest. Inspection through the aortotomy revealed a dilatated aortic annulus (diameter 35 mm) and floppy aortic annulus and leaflets. All leaflets were thin and flail, and had irregular thickening which implied myxomatous degeneration. There was a stiff portion in the left ventricular outflow tract under the noncoronary and right coronary sinus, as a result of the VSD patch. Because we considered that valve-sparing aortic root replacement (VSARR) could be difficult, we performed the Bentall procedure using a 27-mm SJM Masters series Aortic Valved Graft (St. Jude Medical, Cardiology Division Inc., Minnesota), using felt strips in order to reinforce the aortic annulus. After cooling below 20 °C, we performed distal aortic anastomosis using a 28-mm J-Graft Shield Noe (27 mm ) (Japan Lifeline Co. Ltd., Japan) under deep hypothermic circulatory arrest with antegrade cerebral perfusion. After graft-to-graft anastomosis was performed, the patient was easily weaned from the bypass and showed an uneventful course except for the onset of ventricular fibrillation, which was controlled after short-termed assisted circulatory support.

**Fig. 2:**
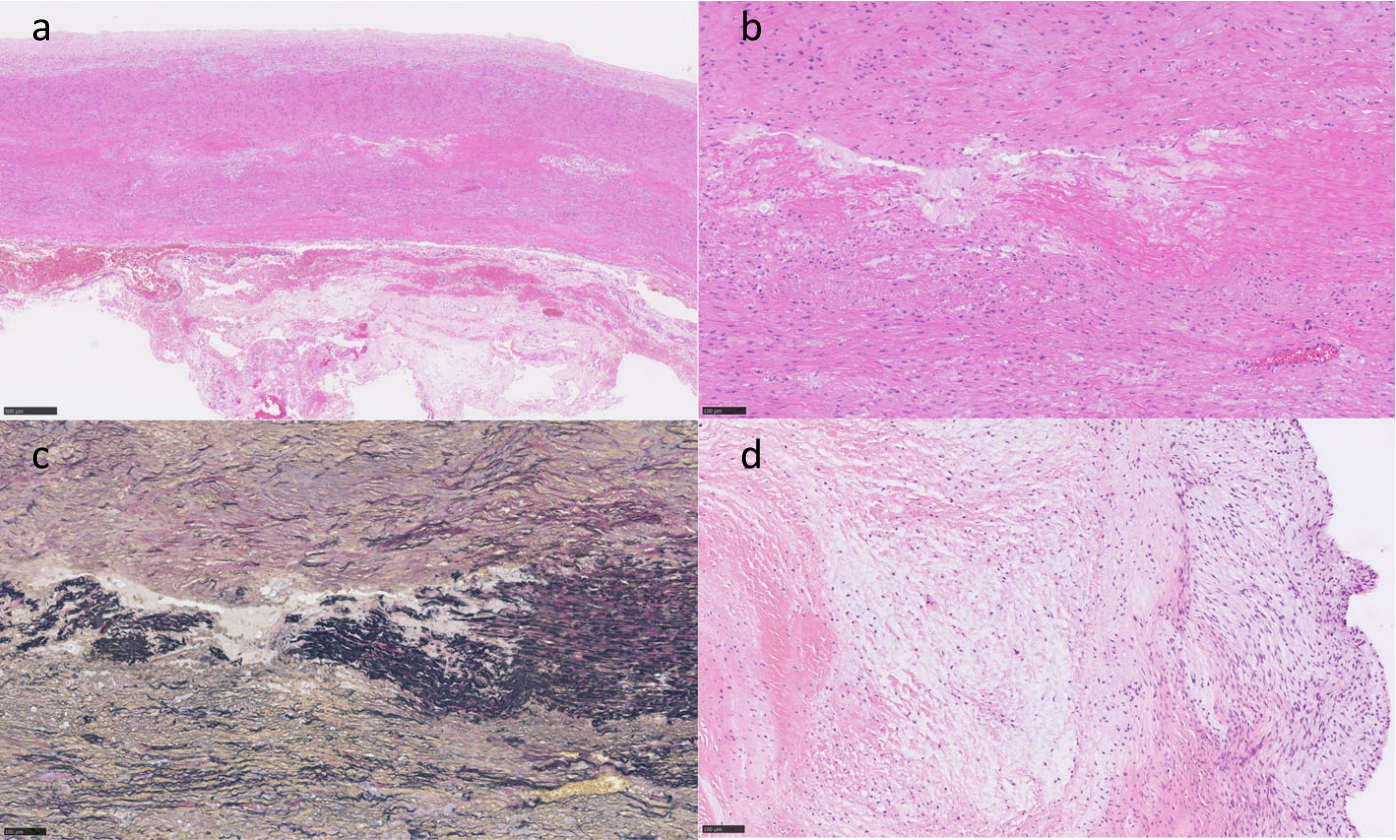
Photomicrographs of the ascending aorta (**a-c**) and aortic valve (**d**) in the patients described in this report. Degeneration of the aorta with significant medial destruction and changes in smooth muscle orientation are observed in addition to accumulation of mucopolysaccharides and collagen deposition (**a** × 50 and **b** × 200, hematoxylin and eosin stain). Fragmentation and loss of elastic lamellae are observed across large areas of the media (**c**, elastica van Gieson stain × 200). Mucoid degeneration of the aortic valve is clearly visualized (**d**, hematoxylin and eosin stain × 200)

Histopathological examination of the ascending aorta specimens revealed cystic medial degeneration with some areas of mucopolysaccharides accumulation, collagen deposition, fragmentation, and loss of elastic lamellae across large areas of the media (Fig. 2a-c). The aortic valve showed mucoid degeneration with fragmentation of elastic fibers (Fig. 2d). The patient’s postoperative course was uneventful, and he was discharged on the 26th postoperative day.

## Discussion

This case received total repair of TOF at 2 years of age, which is not so late age, and underwent ambulatory follow-up without any difficulty including aortic dilatation until 23 years old. We concluded that his aortic diameter had rapidly increased over the last 18 years of adulthood. Unfortunately, he was not evaluated during this time, and his condition remained undetected. Although our patient did not develop aortic dissection, his aortic root showed significant dilatation and histological change of cystic medial degeneration with severe fragmentation and loss of elastic lamellae, which indicate a high-risk patient for aortic dissection. It is unclear that he had histopathological changes in his aortic wall at the first operation due to a lack of histopathological examinations at that time. Aortic dilatation and aneurysm are attributable to an intrinsic mechanism or a secondary effect of increased volume overload of the aorta caused by right-to-left shunting [3–6]. We hypothesize that his aortic dilatation, after adulthood, can be attributed to hypertension, though his history of hypertension is unclear.

Recently, VSARR is reported to be effective for patients with aortic root dilatation and AR not only after repair of congenital heart disease [16, 17], but also with unrepaired TOF [18]. This procedure does not require long-term anti-coagulation therapy because it does not require the use of a mechanical valve and is suitable for young adult patients. Although satisfactory long-term results are reported, the recurrence of AR is one of the problems of this procedure [16, 17]. The presence of more than moderate AR preoperatively has been suggested to be a risk factor for aortic valve durability [16]. The patients with previous repairs of congenital heart disease were considered to have some difficulties such as inherent anatomical abnormality, presence of VSD patch of previous operation, and adhesion due to previous operation. We thought that this case would be a difficult case for VSARR due to the severity of the AR and significant aortic annulus dilatation. Intraoperative inspection revealed that the aortic annulus and leaflets were very thin and floppy. All leaflets were thought to be myxomatous degeneration because of their irregular surface, which was determined with histopathological findings (Fig. 2d). In addition, we could find stiff portions, which seemed to be the VSD patch, just below the floppy annulus. We were afraid that the annulus could be deformed by an annular reduction procedure. We chose the Bentall procedure and reinforced the flail annulus using a felt strip. This frailty seemed unusual in comparison with general annuloaortic ectasia, but it is unclear whether it is a result of extremely dilatated aorta or a characteristic of patients with TOF. If this patient had been diagnosed earlier, when the aortic dilatation and AR were milder, he might be able to have more possibility to receive VSARR.

## Conclusion

We report the case of significant aortic dilatation after TOF repair who showed a rapid increase in aortic diameter after adulthood. Despite the quite low incidence of aortic dissection and very slow growth rate of aortic diameter in general, this case emphasizes the importance of periodic follow-up for proper evaluation of the aorta in patients with TOF, even into adulthood, particularly in male patients with hypertension.

## Data Availability

The data are not available for public access because of patient privacy concerns but are available from the corresponding author on reasonable request.

## Abbreviations

TOF: Tetralogy of Fallot
VSD: Ventricular septal defect
TTE: Transthoracic echocardiography
AR: Aortic valve regurgitation
CT: Computed tomography
RV: Right ventricular
VSARR: Valve-sparing aortic root replacement
